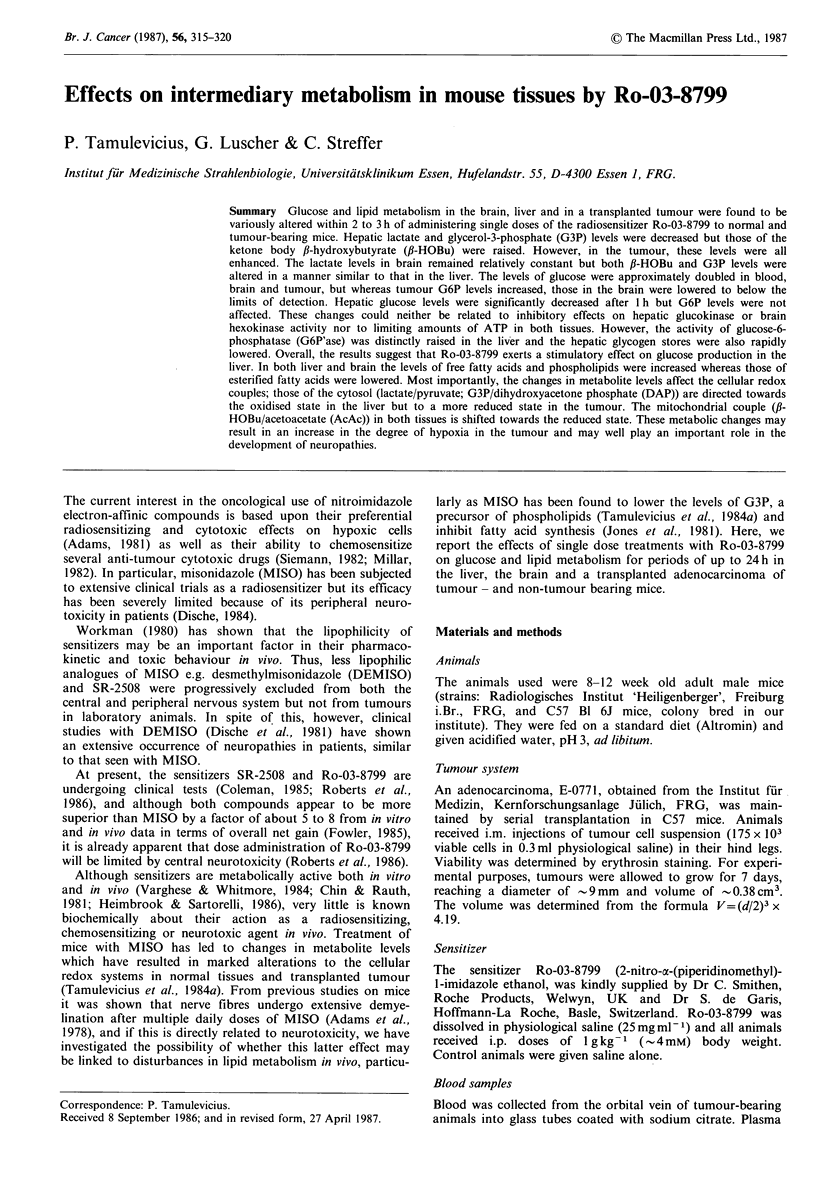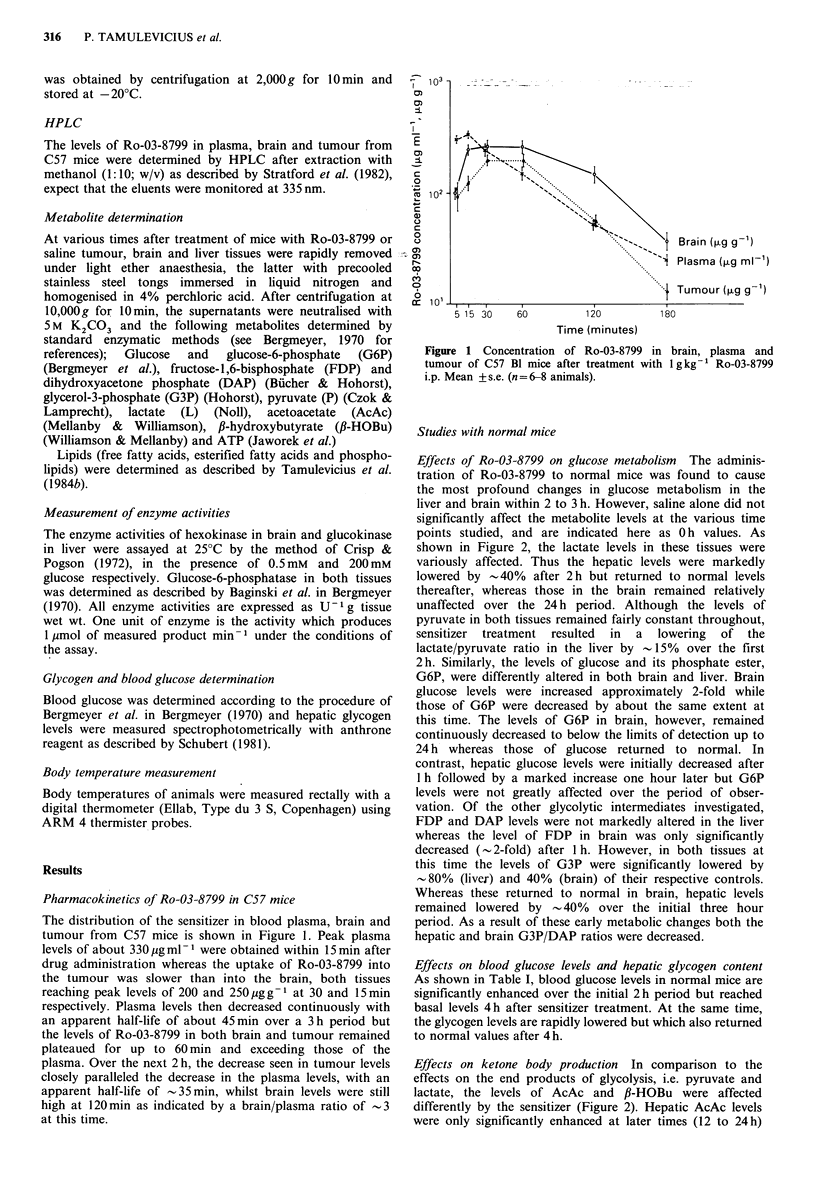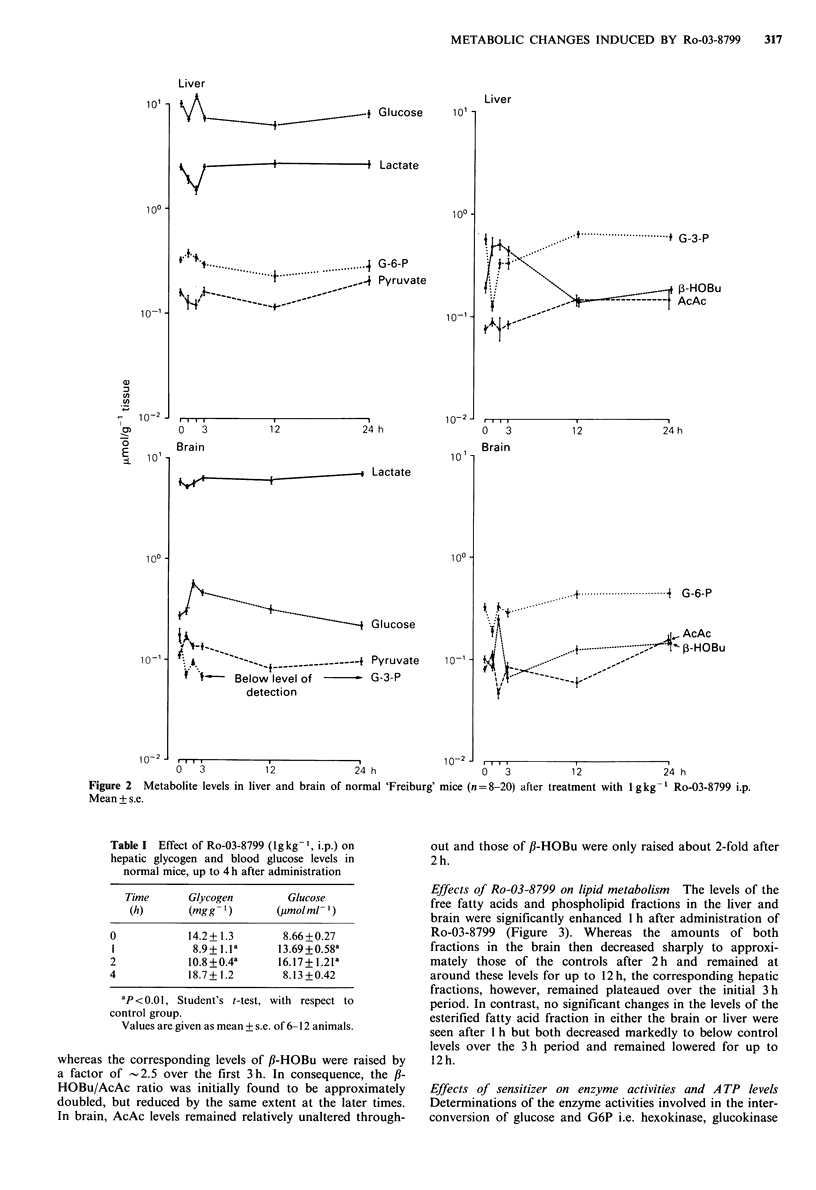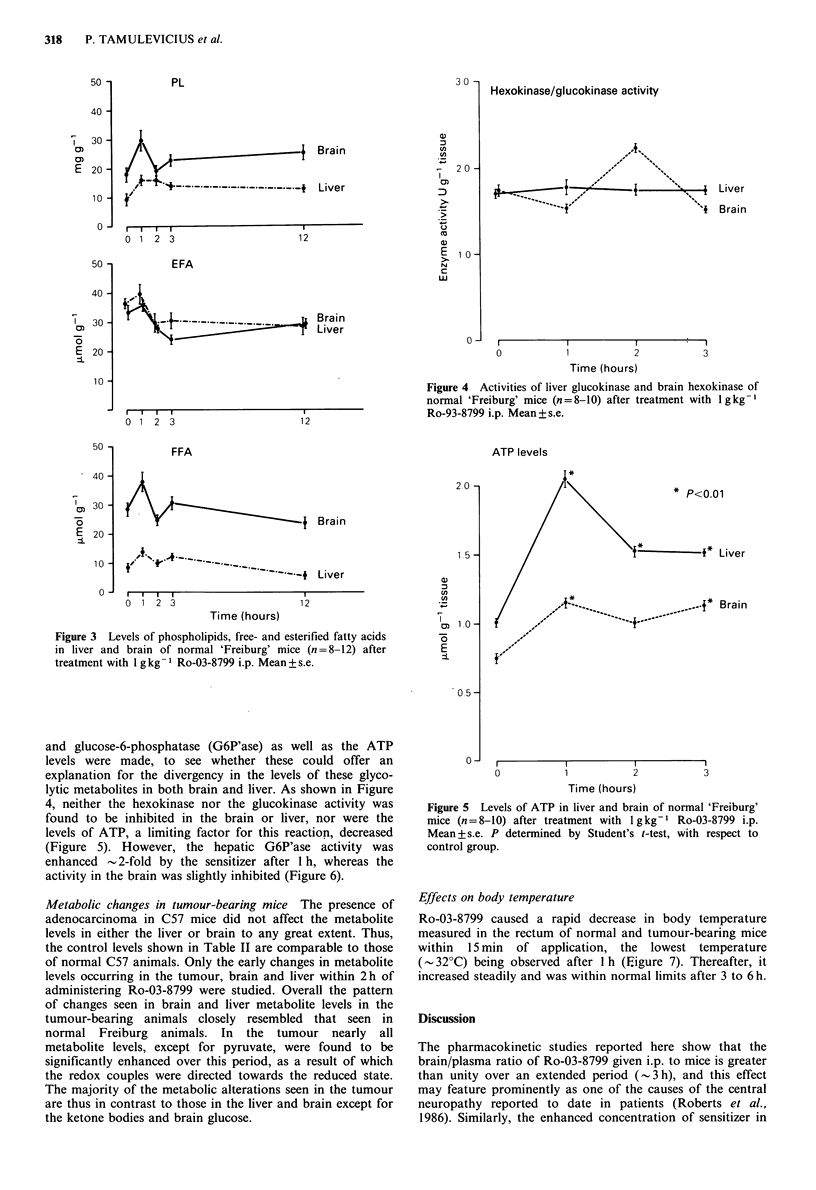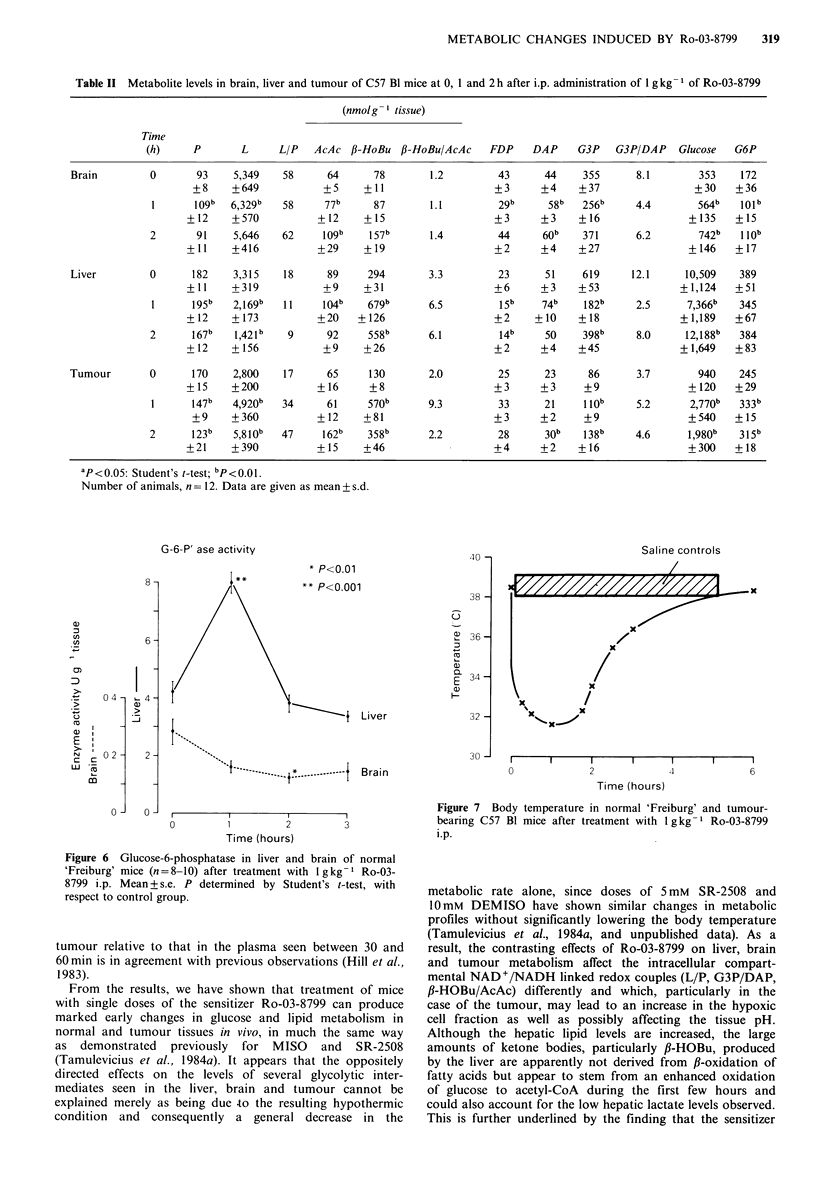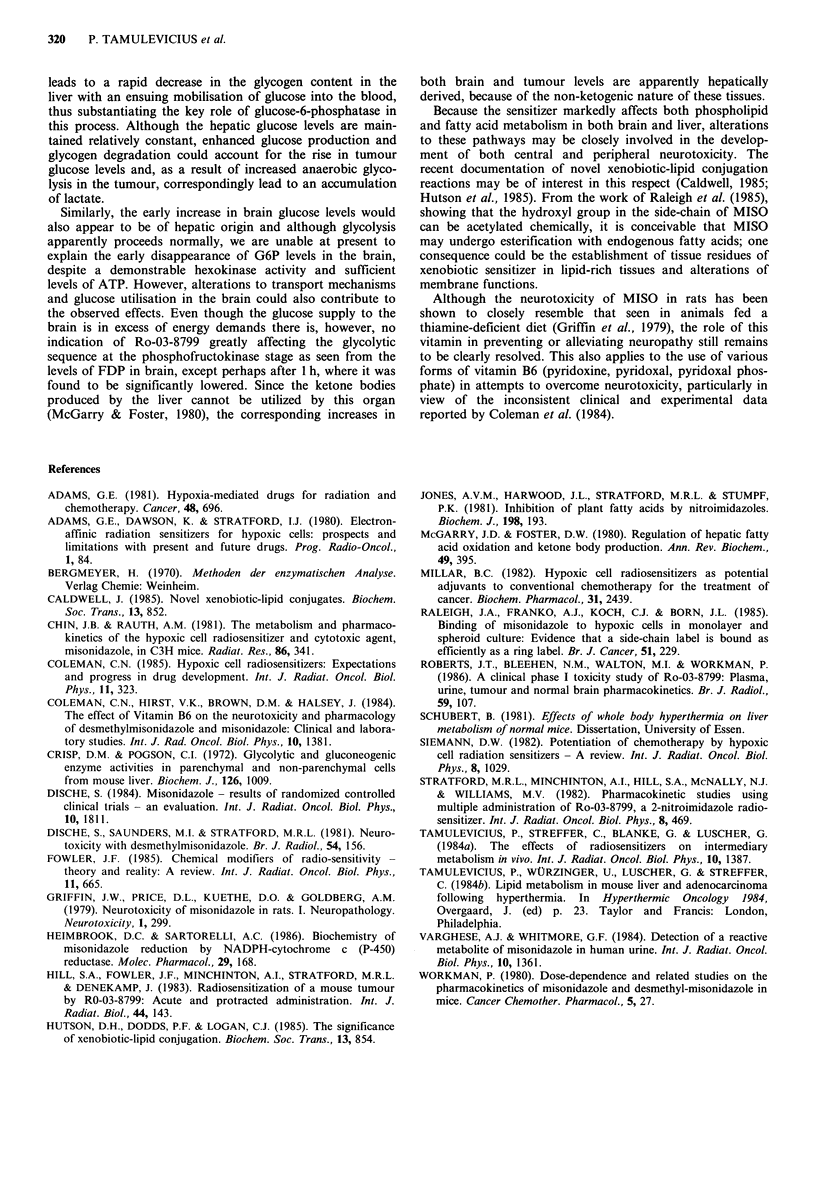# Effects on intermediary metabolism in mouse tissues by Ro-03-8799.

**DOI:** 10.1038/bjc.1987.195

**Published:** 1987-09

**Authors:** P. Tamulevicius, G. Luscher, C. Streffer

**Affiliations:** Institut für Medizinische Strahlenbiologie, Universitätsklinikum Essen, FRG.

## Abstract

Glucose and lipid metabolism in the brain, liver and in a transplanted tumour were found to be variously altered within 2 to 3 h of administering single doses of the radiosensitizer Ro-03-8799 to normal and tumour-bearing mice. Hepatic lactate and glycerol-3-phosphate (G3P) levels were decreased but those of the ketone body beta-hydroxybutyrate (beta-HOBu) were raised. However, in the tumour, these levels were all enhanced. The lactate levels in brain remained relatively constant but both beta-HOBu and G3P levels were altered in a manner similar to that in the liver. The levels of glucose were approximately doubled in blood, brain and tumour, but whereas tumour G6P levels increased, those in the brain were lowered to below the limits of detection. Hepatic glucose levels were significantly decreased after 1 h but G6P levels were not affected. These changes could neither be related to inhibitory effects on hepatic glucokinase or brain hexokinase activity nor to limiting amounts of ATP in both tissues. However, the activity of glucose-6-phosphatase (G6P'ase) was distinctly raised in the liver and the hepatic glycogen stores were also rapidly lowered. Overall, the results suggest that Ro-03-8799 exerts a stimulatory effect on glucose production in the liver. In both liver and brain the levels of free fatty acids and phospholipids were increased whereas those of esterified fatty acids were lowered. Most importantly, the changes in metabolite levels affect the cellular redox couples; those of the cytosol (lactate/pyruvate; G3P/dihydroxyacetone phosphate (DAP] are directed towards the oxidised state in the liver but to a more reduced state in the tumour. The mitochondrial couple (beta-HOBu/acetoacetate (AcAc)) in both tissues is shifted towards the reduced state. These metabolic changes may result in an increase in the degree of hypoxia in the tumour and may well play an important role in the development of neuropathies.


*Br. J. Cancer* (1987), **56**, 315–320 © The Macmillan Press Ltd., 1987

### Effects on intermediary metabolism in mouse tissues by Ro-03-8799

P. Tamulevicius, G. Luscher & C. Streffer

*Institut für Medizinische Strahlenbiologie, Universitätsklinikum Essen, Hufelandstr. 55, D-4300 Essen 1, FRG.*

**Summary** Glucose and lipid metabolism in the brain, liver and in a transplanted tumour were found to be variously altered within 2 to 3 h of administering single doses of the radiosensitizer Ro-03-8799 to normal and tumour-bearing mice. Hepatic lactate and glycerol-3-phosphate (G3P) levels were decreased but those of the ketone body $\beta$-hydroxybutyrate ($\beta$-HOBu) were raised. However, in the tumour, these levels were all enhanced. The lactate levels in brain remained relatively constant but both $\beta$-HOBu and G3P levels were altered in a manner similar to that in the liver. The levels of glucose were approximately doubled in blood, brain and tumour, but whereas tumour G6P levels increased, those in the brain were lowered to below the limits of detection. Hepatic glucose levels were significantly decreased after 1 h but G6P levels were not affected. These changes could neither be related to inhibitory effects on hepatic glucokinase or brain hexokinase activity nor to limiting amounts of ATP in both tissues. However, the activity of glucose-6-phosphatase (G6P'ase) was distinctly raised in the liver and the hepatic glycogen stores were also rapidly lowered. Overall, the results suggest that Ro-03-8799 exerts a stimulatory effect on glucose production in the liver. In both liver and brain the levels of free fatty acids and phospholipids were increased whereas those of esterified fatty acids were lowered. Most importantly, the changes in metabolite levels affect the cellular redox couples; those of the cytosol (lactate/pyruvate; G3P/dihydroxyacetone phosphate (DAP)) are directed towards the oxidised state in the liver but to a more reduced state in the tumour. The mitochondrial couple ($\beta$-HOBu/acetoacetate (AcAc)) in both tissues is shifted towards the reduced state. These metabolic changes may result in an increase in the degree of hypoxia in the tumour and may well play an important role in the development of neuropathies.

The current interest in the oncological use of nitroimidazole electron-affinic compounds is based upon their preferential radiosensitizing and cytotoxic effects on hypoxic cells (Adams, 1981) as well as their ability to chemosensitize several anti-tumour cytotoxic drugs (Siemann, 1982; Millar, 1982). In particular, misonidazole (MISO) has been subjected to extensive clinical trials as a radiosensitizer but its efficacy has been severely limited because of its peripheral neurotoxicity in patients (Dische, 1984).

Workman (1980) has shown that the lipophilicity of sensitizers may be an important factor in their pharmacokinetic and toxic behaviour *in vivo*. Thus, less lipophilic analogues of MISO e.g. desmethylmisonidazole (DEMISO) and SR-2508 were progressively excluded from both the central and peripheral nervous system but not from tumours in laboratory animals. In spite of this, however, clinical studies with DEMISO (Dische *et al.*, 1981) have shown an extensive occurrence of neuropathies in patients, similar to that seen with MISO.

At present, the sensitizers SR-2508 and Ro-03-8799 are undergoing clinical tests (Coleman, 1985; Roberts *et al.*, 1986), and although both compounds appear to be more superior than MISO by a factor of about 5 to 8 from *in vitro* and *in vivo* data in terms of overall net gain (Fowler, 1985), it is already apparent that dose administration of Ro-03-8799 will be limited by central neurotoxicity (Roberts *et al.*, 1986).

Although sensitizers are metabolically active both *in vitro* and *in vivo* (Varghese & Whitmore, 1984; Chin & Rauth, 1981; Heimbrook & Sartorelli, 1986), very little is known biochemically about their action as a radiosensitizing, chemosensitizing or neurotoxic agent *in vivo*. Treatment of mice with MISO has led to changes in metabolite levels which have resulted in marked alterations to the cellular redox systems in normal tissues and transplanted tumour (Tamulevicius *et al.*, 1984*a*). From previous studies on mice it was shown that nerve fibres undergo extensive demyelination after multiple daily doses of MISO (Adams *et al.*, 1978), and if this is directly related to neurotoxicity, we have investigated the possibility of whether this latter effect may be linked to disturbances in lipid metabolism *in vivo*, particularly as MISO has been found to lower the levels of G3P, a precursor of phospholipids (Tamulevicius *et al.*, 1984*a*) and inhibit fatty acid synthesis (Jones *et al.*, 1981). Here, we report the effects of single dose treatments with Ro-03-8799 on glucose and lipid metabolism for periods of up to 24 h in the liver, the brain and a transplanted adenocarcinoma of tumour – and non-tumour bearing mice.

Correspondence: P. Tamulevicius.
Received 8 September 1986; and in revised form, 27 April 1987.

### Materials and methods

### Animals

The animals used were 8–12 week old adult male mice (strains: Radiologisches Institut 'Heiligenberger', Freiburg i.Br., FRG, and C57 Bl 6J mice, colony bred in our institute). They were fed on a standard diet (Altromin) and given acidified water, pH 3, *ad libitum*.

### Tumour system

An adenocarcinoma, E-0771, obtained from the Institut für Medizin, Kernforschungsanlage Jülich, FRG, was maintained by serial transplantation in C57 mice. Animals received i.m. injections of tumour cell suspension ($175 \times 10^3$ viable cells in 0.3 ml physiological saline) in their hind legs. Viability was determined by erythrosin staining. For experimental purposes, tumours were allowed to grow for 7 days, reaching a diameter of $\sim$9 mm and volume of $\sim 0.38\,cm^3$. The volume was determined from the formula $V=(d/2)^3 \times 4.19$.

### Sensitizer

The sensitizer Ro-03-8799 (2-nitro-$\alpha$-(piperidinomethyl)-1-imidazole ethanol, was kindly supplied by Dr C. Smithen, Roche Products, Welwyn, UK and Dr S. de Garis, Hoffmann-La Roche, Basle, Switzerland. Ro-03-8799 was dissolved in physiological saline ($25\,mg\,ml^{-1}$) and all animals received i.p. doses of $1\,g\,kg^{-1}$ ($\sim$4 mM) body weight. Control animals were given saline alone.

### Blood samples

Blood was collected from the orbital vein of tumour-bearing animals into glass tubes coated with sodium citrate. Plasma was obtained by centrifugation at 2,000$g$ for 10 min and stored at −20°C.

*HPLC*

The levels of Ro-03-8799 in plasma, brain and tumour from C57 mice were determined by HPLC after extraction with methanol (1:10; w/v) as described by Stratford *et al.* (1982), expect that the eluents were monitored at 335 nm.

*Metabolite determination*

At various times after treatment of mice with Ro-03-8799 or saline tumour, brain and liver tissues were rapidly removed under light ether anaesthesia, the latter with precooled stainless steel tongs immersed in liquid nitrogen and homogenised in 4% perchloric acid. After centrifugation at 10,000$g$ for 10 min, the supernatants were neutralised with 5 M $K_2CO_3$ and the following metabolites determined by standard enzymatic methods (see Bergmeyer, 1970 for references); Glucose and glucose-6-phosphate (G6P) (Bergmeyer *et al.*), fructose-1,6-bisphosphate (FDP) and dihydroxyacetone phosphate (DAP) (Bücher & Hohorst), glycerol-3-phosphate (G3P) (Hohorst), pyruvate (P) (Czok & Lamprecht), lactate (L) (Noll), acetoacetate (AcAc) (Mellanby & Williamson), $\beta$-hydroxybutyrate ($\beta$-HOBu) (Williamson & Mellanby) and ATP (Jaworek *et al.*)

Lipids (free fatty acids, esterified fatty acids and phospholipids) were determined as described by Tamulevicius *et al.* (1984*b*).

*Measurement of enzyme activities*

The enzyme activities of hexokinase in brain and glucokinase in liver were assayed at 25°C by the method of Crisp & Pogson (1972), in the presence of 0.5 mM and 200 mM glucose respectively. Glucose-6-phosphatase in both tissues was determined as described by Baginski *et al.* in Bergmeyer (1970). All enzyme activities are expressed as $U^{-1}$ g tissue wet wt. One unit of enzyme is the activity which produces 1 $\mu$mol of measured product $min^{-1}$ under the conditions of the assay.

*Glycogen and blood glucose determination*

Blood glucose was determined according to the procedure of Bergmeyer *et al.* in Bergmeyer (1970) and hepatic glycogen levels were measured spectrophotometrically with anthrone reagent as described by Schubert (1981).

*Body temperature measurement*

Body temperatures of animals were measured rectally with a digital thermometer (Ellab, Type du 3 S, Copenhagen) using ARM 4 thermister probes.

### Results

*Pharmacokinetics of Ro-03-8799 in C57 mice*

The distribution of the sensitizer in blood plasma, brain and tumour from C57 mice is shown in Figure 1. Peak plasma levels of about 330 $\mu g\, ml^{-1}$ were obtained within 15 min after drug administration whereas the uptake of Ro-03-8799 into the tumour was slower than into the brain, both tissues reaching peak levels of 200 and 250 $\mu g\, g^{-1}$ at 30 and 15 min respectively. Plasma levels then decreased continuously with an apparent half-life of about 45 min over a 3 h period but the levels of Ro-03-8799 in both brain and tumour remained plateaued for up to 60 min and exceeding those of the plasma. Over the next 2 h, the decrease seen in tumour levels closely paralleled the decrease in the plasma levels, with an apparent half-life of ~35 min, whilst brain levels were still high at 120 min as indicated by a brain/plasma ratio of ~3 at this time.

**Figure 1** Concentration of Ro-03-8799 in brain, plasma and tumour of C57 Bl mice after treatment with 1 g $kg^{-1}$ Ro-03-8799 i.p. Mean ±s.e. ($n$ = 6–8 animals).

*Studies with normal mice*

*Effects of Ro-03-8799 on glucose metabolism* The administration of Ro-03-8799 to normal mice was found to cause the most profound changes in glucose metabolism in the liver and brain within 2 to 3 h. However, saline alone did not significantly affect the metabolite levels at the various time points studied, and are indicated here as 0 h values. As shown in Figure 2, the lactate levels in these tissues were variously affected. Thus the hepatic levels were markedly lowered by ~40% after 2 h but returned to normal levels thereafter, whereas those in the brain remained relatively unaffected over the 24 h period. Although the levels of pyruvate in both tissues remained fairly constant throughout, sensitizer treatment resulted in a lowering of the lactate/pyruvate ratio in the liver by ~15% over the first 2 h. Similarly, the levels of glucose and its phosphate ester, G6P, were differently altered in both brain and liver. Brain glucose levels were increased approximately 2-fold while those of G6P were decreased by about the same extent at this time. The levels of G6P in brain, however, remained continuously decreased to below the limits of detection up to 24 h whereas those of glucose returned to normal. In contrast, hepatic glucose levels were initially decreased after 1 h followed by a marked increase one hour later but G6P levels were not greatly affected over the period of observation. Of the other glycolytic intermediates investigated, FDP and DAP levels were not markedly altered in the liver whereas the level of FDP in brain was only significantly decreased (~2-fold) after 1 h. However, in both tissues at this time the levels of G3P were significantly lowered by ~80% (liver) and 40% (brain) of their respective controls. Whereas these returned to normal in brain, hepatic levels remained lowered by ~40% over the initial three hour period. As a result of these early metabolic changes both the hepatic and brain G3P/DAP ratios were decreased.

*Effects on blood glucose levels and hepatic glycogen content* As shown in Table I, blood glucose levels in normal mice are significantly enhanced over the initial 2 h period but reached basal levels 4 h after sensitizer treatment. At the same time, the glycogen levels are rapidly lowered but which also returned to normal values after 4 h.

*Effects on ketone body production* In comparison to the effects on the end products of glycolysis, i.e. pyruvate and lactate, the levels of AcAc and $\beta$-HOBu were affected differently by the sensitizer (Figure 2). Hepatic AcAc levels were only significantly enhanced at later times (12 to 24 h)

**Figure 2** Metabolite levels in liver and brain of normal 'Freiburg' mice ($n = 8$–$20$) after treatment with $1\,g\,kg^{-1}$ Ro-03-8799 i.p. Mean ± s.e.

**Table I** Effect of Ro-03-8799 ($1g\,kg^{-1}$, i.p.) on hepatic glycogen and blood glucose levels in normal mice, up to 4 h after administration

| *Time* (*h*) | *Glycogen* ($mg\,g^{-1}$) | *Glucose* ($\mu mol\,ml^{-1}$) |
|---|---|---|
| 0 | 14.2 ± 1.3 | 8.66 ± 0.27 |
| 1 | 8.9 ± 1.1[a] | 13.69 ± 0.58[a] |
| 2 | 10.8 ± 0.4[a] | 16.17 ± 1.21[a] |
| 4 | 18.7 ± 1.2 | 8.13 ± 0.42 |

[a]$P < 0.01$, Student's $t$-test, with respect to control group.
Values are given as mean ± s.e. of 6–12 animals.

whereas the corresponding levels of β-HOBu were raised by a factor of ~2.5 over the first 3 h. In consequence, the β-HOBu/AcAc ratio was initially found to be approximately doubled, but reduced by the same extent at the later times. In brain, AcAc levels remained relatively unaltered throughout and those of β-HOBu were only raised about 2-fold after 2 h.

*Effects of Ro-03-8799 on lipid metabolism* The levels of the free fatty acids and phospholipid fractions in the liver and brain were significantly enhanced 1 h after administration of Ro-03-8799 (Figure 3). Whereas the amounts of both fractions in the brain then decreased sharply to approximately those of the controls after 2 h and remained at around these levels for up to 12 h, the corresponding hepatic fractions, however, remained plateaued over the initial 3 h period. In contrast, no significant changes in the levels of the esterified fatty acid fraction in either the brain or liver were seen after 1 h but both decreased markedly to below control levels over the 3 h period and remained lowered for up to 12 h.

*Effects of sensitizer on enzyme activities and ATP levels* Determinations of the enzyme activities involved in the interconversion of glucose and G6P i.e. hexokinase, glucokinase

**Figure 3** Levels of phospholipids, free- and esterified fatty acids in liver and brain of normal 'Freiburg' mice ($n$ = 8–12) after treatment with 1 g kg$^{-1}$ Ro-03-8799 i.p. Mean ± s.e.

**Figure 4** Activities of liver glucokinase and brain hexokinase of normal 'Freiburg' mice ($n$ = 8–10) after treatment with 1 g kg$^{-1}$ Ro-93-8799 i.p. Mean ± s.e.

**Figure 5** Levels of ATP in liver and brain of normal 'Freiburg' mice ($n$ = 8–10) after treatment with 1 g kg$^{-1}$ Ro-03-8799 i.p. Mean ± s.e. $P$ determined by Student's $t$-test, with respect to control group.

and glucose-6-phosphatase (G6P'ase) as well as the ATP levels were made, to see whether these could offer an explanation for the divergency in the levels of these glycolytic metabolites in both brain and liver. As shown in Figure 4, neither the hexokinase nor the glucokinase activity was found to be inhibited in the brain or liver, nor were the levels of ATP, a limiting factor for this reaction, decreased (Figure 5). However, the hepatic G6P'ase activity was enhanced ~2-fold by the sensitizer after 1 h, whereas the activity in the brain was slightly inhibited (Figure 6).

*Metabolic changes in tumour-bearing mice* The presence of adenocarcinoma in C57 mice did not affect the metabolite levels in either the liver or brain to any great extent. Thus, the control levels shown in Table II are comparable to those of normal C57 animals. Only the early changes in metabolite levels occurring in the tumour, brain and liver within 2 h of administering Ro-03-8799 were studied. Overall the pattern of changes seen in brain and liver metabolite levels in the tumour-bearing animals closely resembled that seen in normal Freiburg animals. In the tumour nearly all metabolite levels, except for pyruvate, were found to be significantly enhanced over this period, as a result of which the redox couples were directed towards the reduced state. The majority of the metabolic alterations seen in the tumour are thus in contrast to those in the liver and brain except for the ketone bodies and brain glucose.

*Effects on body temperature*

Ro-03-8799 caused a rapid decrease in body temperature measured in the rectum of normal and tumour-bearing mice within 15 min of application, the lowest temperature (~32°C) being observed after 1 h (Figure 7). Thereafter, it increased steadily and was within normal limits after 3 to 6 h.

### Discussion

The pharmacokinetic studies reported here show that the brain/plasma ratio of Ro-03-8799 given i.p. to mice is greater than unity over an extended period (~3 h), and this effect may feature prominently as one of the causes of the central neuropathy reported to date in patients (Roberts *et al.*, 1986). Similarly, the enhanced concentration of sensitizer in

**Table II** Metabolite levels in brain, liver and tumour of C57 Bl mice at 0, 1 and 2 h after i.p. administration of 1 g kg$^{-1}$ of Ro-03-8799

| | Time (h) | P | L | L/P | AcAc | β-HoBu | β-HoBu/AcAc | FDP | DAP | G3P | G3P/DAP | Glucose | G6P |
|---|---|---|---|---|---|---|---|---|---|---|---|---|---|
| | | (nmol g$^{-1}$ tissue) | | | | | | | | | | | |
| Brain | 0 | 93 ±8 | 5,349 ±649 | 58 | 64 ±5 | 78 ±11 | 1.2 | 43 ±3 | 44 ±4 | 355 ±37 | 8.1 | 353 ±30 | 172 ±36 |
| | 1 | 109[b] ±12 | 6,329[b] ±570 | 58 | 77[b] ±12 | 87 ±15 | 1.1 | 29[b] ±3 | 58[b] ±3 | 256[b] ±16 | 4.4 | 564[b] ±135 | 101[b] ±15 |
| | 2 | 91 ±11 | 5,646 ±416 | 62 | 109[b] ±29 | 157[b] ±19 | 1.4 | 44 ±2 | 60[b] ±4 | 371 ±27 | 6.2 | 742[b] ±146 | 110[b] ±17 |
| Liver | 0 | 182 ±11 | 3,315 ±319 | 18 | 89 ±9 | 294 ±31 | 3.3 | 23 ±6 | 51 ±3 | 619 ±53 | 12.1 | 10,509 ±1,124 | 389 ±51 |
| | 1 | 195[b] ±12 | 2,169[b] ±173 | 11 | 104[b] ±20 | 679[b] ±126 | 6.5 | 15[b] ±2 | 74[b] ±10 | 182[b] ±18 | 2.5 | 7,366[b] ±1,189 | 345 ±67 |
| | 2 | 167[b] ±12 | 1,421[b] ±156 | 9 | 92 ±9 | 558[b] ±26 | 6.1 | 14[b] ±2 | 50 ±4 | 398[b] ±45 | 8.0 | 12,188[b] ±1,649 | 384 ±83 |
| Tumour | 0 | 170 ±15 | 2,800 ±200 | 17 | 65 ±16 | 130 ±8 | 2.0 | 25 ±3 | 23 ±3 | 86 ±9 | 3.7 | 940 ±120 | 245 ±29 |
| | 1 | 147[b] ±9 | 4,920[b] ±360 | 34 | 61 ±12 | 570[b] ±81 | 9.3 | 33 ±3 | 21 ±2 | 110[b] ±9 | 5.2 | 2,770[b] ±540 | 333[b] ±15 |
| | 2 | 123[b] ±21 | 5,810[b] ±390 | 47 | 162[b] ±15 | 358[b] ±46 | 2.2 | 28 ±4 | 30[b] ±2 | 138[b] ±16 | 4.6 | 1,980[b] ±300 | 315[b] ±18 |

[a]$P<0.05$: Student's *t*-test; [b]$P<0.01$.
Number of animals, $n=12$. Data are given as mean ± s.d.

**Figure 6** Glucose-6-phosphatase in liver and brain of normal 'Freiburg' mice ($n=8$–10) after treatment with 1 g kg$^{-1}$ Ro-03-8799 i.p. Mean ± s.e. *P* determined by Student's *t*-test, with respect to control group.

**Figure 7** Body temperature in normal 'Freiburg' and tumour-bearing C57 Bl mice after treatment with 1 g kg$^{-1}$ Ro-03-8799 i.p.

tumour relative to that in the plasma seen between 30 and 60 min is in agreement with previous observations (Hill *et al.*, 1983).

From the results, we have shown that treatment of mice with single doses of the sensitizer Ro-03-8799 can produce marked early changes in glucose and lipid metabolism in normal and tumour tissues *in vivo*, in much the same way as demonstrated previously for MISO and SR-2508 (Tamulevicius *et al.*, 1984*a*). It appears that the oppositely directed effects on the levels of several glycolytic intermediates seen in the liver, brain and tumour cannot be explained merely as being due to the resulting hypothermic condition and consequently a general decrease in the metabolic rate alone, since doses of 5 mM SR-2508 and 10 mM DEMISO have shown similar changes in metabolic profiles without significantly lowering the body temperature (Tamulevicius *et al.*, 1984*a*, and unpublished data). As a result, the contrasting effects of Ro-03-8799 on liver, brain and tumour metabolism affect the intracellular compartmental $NAD^+$/NADH linked redox couples (L/P, G3P/DAP, β-HOBu/AcAc) differently and which, particularly in the case of the tumour, may lead to an increase in the hypoxic cell fraction as well as possibly affecting the tissue pH. Although the hepatic lipid levels are increased, the large amounts of ketone bodies, particularly β-HOBu, produced by the liver are apparently not derived from β-oxidation of fatty acids but appear to stem from an enhanced oxidation of glucose to acetyl-CoA during the first few hours and could also account for the low hepatic lactate levels observed. This is further underlined by the finding that the sensitizer leads to a rapid decrease in the glycogen content in the liver with an ensuing mobilisation of glucose into the blood, thus substantiating the key role of glucose-6-phosphatase in this process. Although the hepatic glucose levels are maintained relatively constant, enhanced glucose production and glycogen degradation could account for the rise in tumour glucose levels and, as a result of increased anaerobic glycolysis in the tumour, correspondingly lead to an accumulation of lactate.

Similarly, the early increase in brain glucose levels would also appear to be of hepatic origin and although glycolysis apparently proceeds normally, we are unable at present to explain the early disappearance of G6P levels in the brain, despite a demonstrable hexokinase activity and sufficient levels of ATP. However, alterations to transport mechanisms and glucose utilisation in the brain could also contribute to the observed effects. Even though the glucose supply to the brain is in excess of energy demands there is, however, no indication of Ro-03-8799 greatly affecting the glycolytic sequence at the phosphofructokinase stage as seen from the levels of FDP in brain, except perhaps after 1 h, where it was found to be significantly lowered. Since the ketone bodies produced by the liver cannot be utilized by this organ (McGarry & Foster, 1980), the corresponding increases in both brain and tumour levels are apparently hepatically derived, because of the non-ketogenic nature of these tissues.

Because the sensitizer markedly affects both phospholipid and fatty acid metabolism in both brain and liver, alterations to these pathways may be closely involved in the development of both central and peripheral neurotoxicity. The recent documentation of novel xenobiotic-lipid conjugation reactions may be of interest in this respect (Caldwell, 1985; Hutson *et al.*, 1985). From the work of Raleigh *et al.* (1985), showing that the hydroxyl group in the side-chain of MISO can be acetylated chemically, it is conceivable that MISO may undergo esterification with endogenous fatty acids; one consequence could be the establishment of tissue residues of xenobiotic sensitizer in lipid-rich tissues and alterations of membrane functions.

Although the neurotoxicity of MISO in rats has been shown to closely resemble that seen in animals fed a thiamine-deficient diet (Griffin *et al.*, 1979), the role of this vitamin in preventing or alleviating neuropathy still remains to be clearly resolved. This also applies to the use of various forms of vitamin B6 (pyridoxine, pyridoxal, pyridoxal phosphate) in attempts to overcome neurotoxicity, particularly in view of the inconsistent clinical and experimental data reported by Coleman *et al.* (1984).

### References

ADAMS, G.E. (1981). Hypoxia-mediated drugs for radiation and chemotherapy. *Cancer*, **48**, 696.

ADAMS, G.E., DAWSON, K. & STRATFORD, I.J. (1980). Electron-affinic radiation sensitizers for hypoxic cells: prospects and limitations with present and future drugs. *Prog. Radio-Oncol.*, **1**, 84.

BERGMEYER, H. (1970). *Methoden der enzymatischen Analyse*. Verlag Chemie: Weinheim.

CALDWELL, J. (1985). Novel xenobiotic-lipid conjugates. *Biochem. Soc. Trans.*, **13**, 852.

CHIN, J.B. & RAUTH, A.M. (1981). The metabolism and pharmacokinetics of the hypoxic cell radiosensitizer and cytotoxic agent, misonidazole, in C3H mice. *Radiat. Res.*, **86**, 341.

COLEMAN, C.N. (1985). Hypoxic cell radiosensitizers: Expectations and progress in drug development. *Int. J. Radiat. Oncol. Biol. Phys.*, **11**, 323.

COLEMAN, C.N., HIRST, V.K., BROWN, D.M. & HALSEY, J. (1984). The effect of Vitamin B6 on the neurotoxicity and pharmacology of desmethylmisonidazole and misonidazole: Clinical and laboratory studies. *Int. J. Rad. Oncol. Biol. Phys.*, **10**, 1381.

CRISP, D.M. & POGSON, C.I. (1972). Glycolytic and gluconeogenic enzyme activities in parenchymal and non-parenchymal cells from mouse liver. *Biochem. J.*, **126**, 1009.

DISCHE, S. (1984). Misonidazole – results of randomized controlled clinical trials – an evaluation. *Int. J. Radiat. Oncol. Biol. Phys.*, **10**, 1811.

DISCHE, S., SAUNDERS, M.I. & STRATFORD, M.R.L. (1981). Neurotoxicity with desmethylmisonidazole. *Br. J. Radiol.*, **54**, 156.

FOWLER, J.F. (1985). Chemical modifiers of radio-sensitivity – theory and reality: A review. *Int. J. Radiat. Oncol. Biol. Phys.*, **11**, 665.

GRIFFIN, J.W., PRICE, D.L., KUETHE, D.O. & GOLDBERG, A.M. (1979). Neurotoxicity of misonidazole in rats. I. Neuropathology. *Neurotoxicity*, **1**, 299.

HEIMBROOK, D.C. & SARTORELLI, A.C. (1986). Biochemistry of misonidazole reduction by NADPH-cytochrome c (P-450) reductase. *Molec. Pharmacol.*, **29**, 168.

HILL, S.A., FOWLER, J.F., MINCHINTON, A.I., STRATFORD, M.R.L. & DENEKAMP, J. (1983). Radiosensitization of a mouse tumour by R0-03-8799: Acute and protracted administration. *Int. J. Radiat. Biol.*, **44**, 143.

HUTSON, D.H., DODDS, P.F. & LOGAN, C.J. (1985). The significance of xenobiotic-lipid conjugation. *Biochem. Soc. Trans.*, **13**, 854.

JONES, A.V.M., HARWOOD, J.L., STRATFORD, M.R.L. & STUMPF, P.K. (1981). Inhibition of plant fatty acids by nitroimidazoles. *Biochem. J.*, **198**, 193.

McGARRY, J.D. & FOSTER, D.W. (1980). Regulation of hepatic fatty acid oxidation and ketone body production. *Ann. Rev. Biochem.*, **49**, 395.

MILLAR, B.C. (1982). Hypoxic cell radiosensitizers as potential adjuvants to conventional chemotherapy for the treatment of cancer. *Biochem. Pharmacol.*, **31**, 2439.

RALEIGH, J.A., FRANKO, A.J., KOCH, C.J. & BORN, J.L. (1985). Binding of misonidazole to hypoxic cells in monolayer and spheroid culture: Evidence that a side-chain label is bound as efficiently as a ring label. *Br. J. Cancer*, **51**, 229.

ROBERTS, J.T., BLEEHEN, N.M., WALTON, M.I. & WORKMAN, P. (1986). A clinical phase I toxicity study of Ro-03-8799: Plasma, urine, tumour and normal brain pharmacokinetics. *Br. J. Radiol.*, **59**, 107.

SCHUBERT, B. (1981). *Effects of whole body hyperthermia on liver metabolism of normal mice*. Dissertation, University of Essen.

SIEMANN, D.W. (1982). Potentiation of chemotherapy by hypoxic cell radiation sensitizers – A review. *Int. J. Radiat. Oncol. Biol. Phys.*, **8**, 1029.

STRATFORD, M.R.L., MINCHINTON, A.I., HILL, S.A., McNALLY, N.J. & WILLIAMS, M.V. (1982). Pharmacokinetic studies using multiple administration of Ro-03-8799, a 2-nitroimidazole radiosensitizer. *Int. J. Radiat. Oncol. Biol. Phys.*, **8**, 469.

TAMULEVICIUS, P., STREFFER, C., BLANKE, G. & LUSCHER, G. (1984*a*). The effects of radiosensitizers on intermediary metabolism *in vivo*. *Int. J. Radiat. Oncol. Biol. Phys.*, **10**, 1387.

TAMULEVICIUS, P., WÜRZINGER, U., LUSCHER, G. & STREFFER, C. (1984*b*). Lipid metabolism in mouse liver and adenocarcinoma following hyperthermia. In *Hyperthermic Oncology 1984*, Overgaard, J. (ed) p. 23. Taylor and Francis: London, Philadelphia.

VARGHESE, A.J. & WHITMORE, G.F. (1984). Detection of a reactive metabolite of misonidazole in human urine. *Int. J. Radiat. Oncol. Biol. Phys.*, **10**, 1361.

WORKMAN, P. (1980). Dose-dependence and related studies on the pharmacokinetics of misonidazole and desmethyl-misonidazole in mice. *Cancer Chemother. Pharmacol.*, **5**, 27.